# A Fast Transient Response Capacitor-Less LDO with Transient Enhancement Technology

**DOI:** 10.3390/mi15030299

**Published:** 2024-02-22

**Authors:** Chufan Chen, Mengyuan Sun, Leiyi Wang, Teng Huang, Min Xu

**Affiliations:** 1 State Key Laboratory of ASIC and System, School of Microelectronics, Fudan University, Shanghai 200433, China; 21212020002@m.fudan.edu.cn (C.C.); 20212020003@fudan.edu.cn (M.S.); lywang2018@stu.suda.edu.cn (L.W.); 21212020005@m.fudan.edu.cn (T.H.); 2 School of Electronic and Information Engineering, Soochow University, Suzhou 215006, China

**Keywords:** low dropout regulator, capacitor-less, transient enhancement circuit, super source follower

## Abstract

This paper proposes a fast transient load response capacitor-less low-dropout regulator (CL-LDO) for digital analog hybrid circuits in the 180 nm process, capable of converting input voltages from 1.2 V to 1.8 V into an output voltage of 1 V. The design incorporates a rail-to-rail input and push–pull output (RIPO) amplifier to enhance the gain while satisfying the requirement for low power consumption. A super source follower buffer (SSFB) with internal stability is introduced to ensure loop stability. The proposed structure ensures the steady-state performance of the LDO without an on-chip capacitor. The auxiliary circuit, or transient enhancement circuit, does not compromise the steady-state stability and effectively enhances the transient performance during sudden load current steps. The proposed LDO consumes a quiescent current of 47 µA and achieves 25 µV/mA load regulation with a load current ranging from 0 to 20 mA. The simulation results demonstrate that a settling time of 0.2 µs is achieved for load steps ranging from 0 mA to 20 mA, while a settling time of 0.5 µs is attained for load steps ranging from 20 mA to 0 mA, with an edge time of 0.1 µs.

## 1. Introduction

Power management ICs (PMICs) are essential components in electronic devices that require efficient power management [1]. Low-dropout regulators (LDOs) are preferred over other voltage regulators due to their low noise, low ripple, low quiescent current and high power supply rejection ratio (PSRR) [2,3,4,5]. To satisfy the different voltage regulation requirements of various modules in a single system-on-chip (SOC), multiple voltage regulators can be integrated on the same chip. This approach can reduce the power dissipation and improve the overall efficiency of the system. In SoC designs, LDOs are commonly used to supply power to analog or mixed-signal modules, which are particularly sensitive to noise and voltage fluctuations [6,7,8]. However, the traditional LDO architecture relies on a large capacitor, which occupies a large area and reduces circuit integration. Removal of this large off-chip capacitor will inevitably degrade the performance requirements of the LDO, especially stability, transient response and PSRR. Therefore, in recent years, capacitor-less LDOs (CL-LDOs) have been widely studied and reported [9,10,11].

A series of techniques have been proposed to improve the transient response of CL-LDO [12,13,14,15,16,17]. Using a flipped voltage follower (FVF) to separate the dominant poles in conventional LDO is one of the most popular methods. The FVF and overshoot detection circuit used in [12] reduce the overshoot/undershoot voltages of LDO and achieve fast settling times during load steps. The push–pull amplifier is a kind of architecture that can be used to provide a fast response to load and line transients [13,15]. The LDO with Class-AB OTA in [13] provides not only a fast response to load and line transients, but also handles a wide range of load capacitors, while the push–pull output stage-based LDO in [15] can achieve a 2.7 µs settling time with the load current switching from 100 pA to 100 mA. A dynamic biasing technique is widely used, whereby the bias current of the LDO is adjusted based on the load current. This can improve the efficiency of the LDO and reduce the power dissipation. To enhance both the transient and stability, Li et al. proposed a CL-LDO based on dual-active feedback frequency compensation that ultimately guarantees stable operation in a load range of 0 to 100 mA [14]. In [17], the authors use modified Miller compensation with the insertion of a sensor amplifier stage to inject more transient current in the biasing circuit. This method feeds the regulator to rapidly charge the power PMOS gate capacitance and improves the fast transient response.

This work proposes a novel CL-LDO circuit with a fast transient response. Section 2 demonstrates the complete architecture with a rail-to-rail input, push–pull output (RIPO) two-stage amplifier and a super source follower buffer (SSFB) and analyzes the stability and transient response. Section 3 presents a design example, validated through simulation results, and compares this work with others. Section 4 summarizes the conclusion. 

## 2. Proposed CL-LDO Architecture with RIPO and SSFB

### 2.1. Conventional Topology of LDO

**Figure 1:**
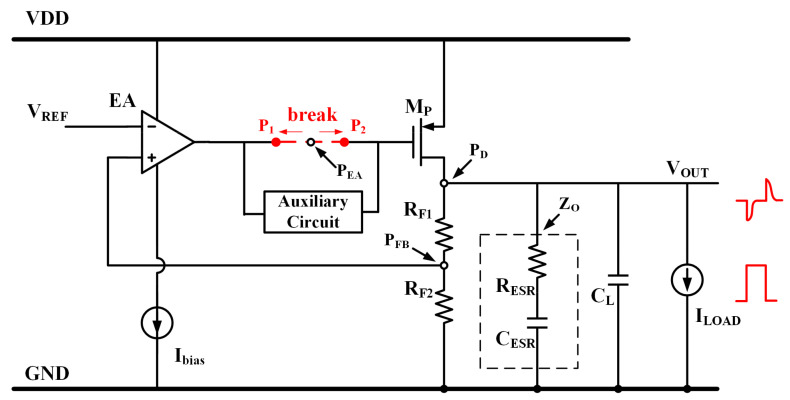
Conventional architecture of LDO.

The traditional LDO topology with an off-chip capacitor depicted in Figure 1 exhibits three poles and a left-half-plane zero without the need for an auxiliary circuit. The stability of this system is ensured by the presence of a left-half-plane zero, which is generated by the output capacitor with a capacitance in the microfarad range and its equivalent resistance. Additionally, Equation (1) establishes that the dominant pole is positioned at the output node. The remaining two poles are positioned at the output of the amplifier and feedback resistance. The removal of this bully output capacitor poses a greater challenge to the stability. To enhance the stability, compensation capacitors are added in the auxiliary circuit.

(1) $$P_{1}=\frac{1}{\left[\left(R_{1}+R_{2}\right)\left|\left|R_{L}\right|\right|r_{op}\right]\left(C_{L}+C_{O}\right)}$$

The spike output voltage in the LDO when there are sudden changes in the load current from other cells is also depicted in Figure 1. The unity gain frequency (UGF) is one of the most essential factors of transient performance. To achieve a large UGF, prior studies have proposed an architecture incorporating a buffer as the auxiliary circuit to decouple the high impedance from the EA’s output and the large capacitance from the M_P_’s input. Additionally, the response time *T_R_* [12] of the LDO can be approximated as Equation (2):

(2) $$T_{R}\approx \frac{1}{\mathrm{BW}}+C_{\mathrm{par}}\frac{\Delta V_{G}}{I_{G}}$$

where BW denotes the loop bandwidth, *C_par_* represents the parasitic cap, Δ*V_G_* refers to the required voltage change and *I_G_* represents the slewing current at M_P_’s gate. The buffer with a low output resistance can offer a high slewing current to rapidly respond to the load step.

### 2.2. Proposed RIPO and SSFB

**Figure 2:**
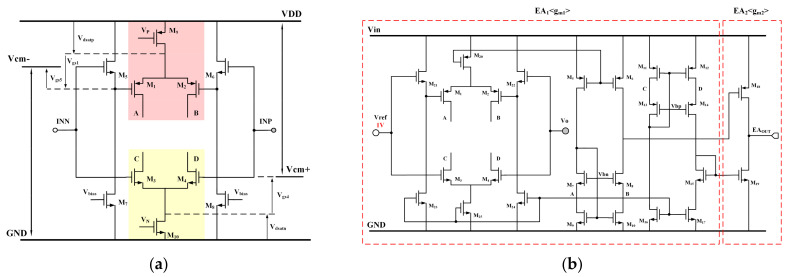
Structures of (**a**) input stage of RIPO amplifier, (**b**) complete schematic of RIPO amplifier.

A rail-to-rail input of the RIPO amplifier is shown in Figure 2a. The PMOS input pair M_1_–M_2_ is utilized to achieve the negative supply rail, while the NMOS input pair M_3_–M_4_ is employed to reach the positive supply rail. The transistors M_5_ to M_8_ are used as level shifters for the PMOS input pair, thereby expanding the negative input range to ensure that the PMOS input pair operates in the saturation region. The tail currents of two complementary input pairs are supported by M_9_ and M_10_. The positive supply rail extends from *Vcm*+ to *VDD*, while the negative supply rail spans from GND to *Vcm*−. The expressions for *Vcm*+ and *Vcm*− are represented by Equation (3) and Equation (4), respectively:

(3) $$Vcm+=V_{dsatn}+V_{gs4}$$

(4) $$Vcm-=VDD-V_{dsatp}-V_{gs1}+V_{gs5}\approx VDD-V_{dsatp}$$

where *V_dsatn_* and *V_dsatp_* are the minimum drain–source voltages that ensure M_9_ and M_10_ operate as current sources. When *VDD* > *V_gs_*_4_ + *V_dsatn_* + *V_dsatp_* = *V_gs_* + 2*V_dsat_*, the input range is obviously from 0 to *VDD*. The complete RIPO amplifier circuit is depicted in Figure 2b. It is based on the compact cascode amplifier (EA_1_) with rail-to-rail input, where the gate voltage of EA_2_ is sourced from the output of EA_1_. When the voltage of INP, the negative input of EA_1_, increases, the voltages of b and d decrease simultaneously. In this case, both gate voltages of M_24_ and M_25_ are reversed from INP and in phase at INN. So, the output stage, EA_2_, functions as a push–pull amplifier in the RIPO circuit. The stability analysis of this RIPO amplifier configuration with CC_1_ is addressed in Section 2.3.

**Figure 3:**
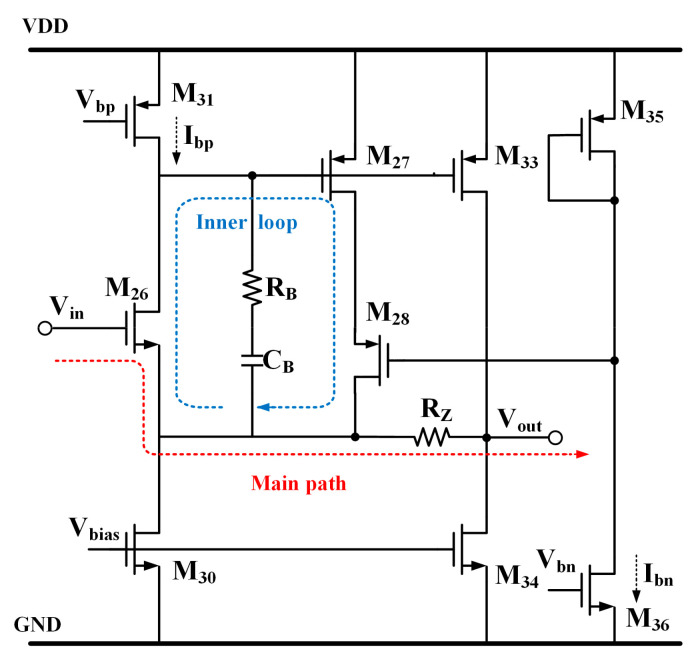
Schematic of super source follower buffer.

The proposed SSFB added as an auxiliary circuit is shown in Figure 3. The core components of the SSFB are M_26_–M_28_, where the M_26_ is used as the source follower, while M_27_ and M_28_ serve to enhance the following capability. The primary signal transmission pathway involves the passage of signals from the gate of M_26_ through resistor Rz to reach the output terminal. The node at the gate of M_27_ is a high impedance node. Generally, the stability of a buffer solely based on this main signal path is not taken into consideration. However, it should be noted that the proposed buffer also incorporates an inner loop, which may cause stability problems. To deal with the stability issue, compensation is achieved by incorporating capacitor C_B_ and resistors R_B_ R_Z_. A detailed analysis is provided in Section 2.3. The transistors M_31_ and M_36_ are used as current sources to supply the static operating currents I_bp_ and I_bn_, respectively. The ratio of I_bp_ to I_bn_ is set at 1:4, with I_bp_ biased at 0.25 µA and I_bn_ biased at 1 µA.

### 2.3. Stability Analysis

**Figure 4:**
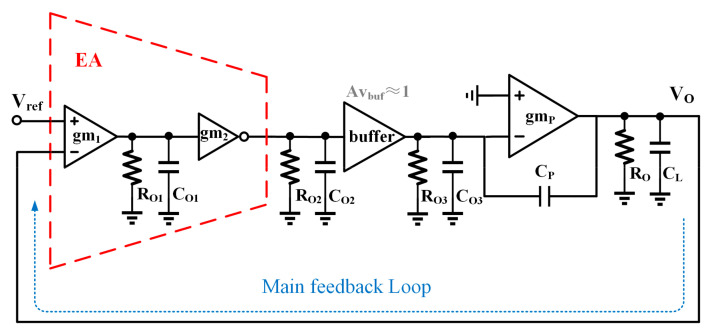
Small-signal modeling of proposed CL-LDO.

The simplified structure of the whole CL-LDO with RIPO and SSFB is illustrated in Figure 4. The main feedback loop of the LDO consistently employs linear feedback, and this paper uses the unit negative feedback. Considering that the transfer function of the buffer is close to unity except at high frequencies, and considering its large input impedance, we temporarily substitute it with *A_vbuf_* ≈ 1 when analyzing the frequency response of CL-LDO. This is discussed separately later. The pole inside EA, which is at an extremely high frequency due to the small parasitic capacitance *C_O_*_1_ and is composed of the output of E_A1_ and input of E_A2_, is disregarded in the frequency response analysis of the proposed CL-LDO. *R_O_*_2_ and *C_O_*_2_ stand for the output resistance of EA and the input parasitic capacitor of the buffer, respectively. *C_O_*_3_ comprises the gate–source capacitor (*C_gs_*) of the power PMOS M_P_ and the output parasitic capacitor of the buffer. Considering that the parasitic capacitance is significantly smaller than *C_gs_* by several orders of magnitude, it can be approximated that *C_O_*_3_ is approximately equal to *C_gs_*. *R_O_*_3_ is equal to the output resistance of the super source buffer, which is extremely small. *C_gP_* consists of the Miller compensation capacitor, *C_P_*, and the gate–drain capacitor (*C_gP_*) of M_P_. The resistance *R_O_* denotes the equivalent output resistance, which is influenced by the load current, while *C_L_* represents the load capacitor. The *A_v_*(*s*) is given by Equations (5)–(9).

(5) $$A_{v}\left(s\right)=\frac{V_{out}\left(s\right)}{V_{in}\left(s\right)}\approx \frac{A_{dc}\left(1-\frac{sC_{gP}}{g_{mP}}\right)}{\left(1+\frac{s}{p_{1}}\right)\left(1+\frac{s}{p_{2}}\right)\left(1+\frac{s}{p_{3}}\right)}$$

(6) $$A_{dc}={g_{m}}_{1}R_{O1}\times {g_{m}}_{2}R_{O2}\times A_{vbuf}\times {g_{m}}_{P}R_{O}$$

(7) $$P_{1}\approx \frac{1}{R_{O3}\left(C_{O3}+\left(1+{g_{m}}_{P}R_{O}\right)C_{gP}\right)}$$

(8) $$P_{2}\approx \frac{\left(1+{g_{m}}_{P}R_{O}\right)C_{P}+C_{O3}}{R_{O}\left(C_{L}C_{P}+C_{P}C_{O3}+C_{L}C_{O3}\right)}$$

(9) $$P_{3}=\frac{1}{R_{O2}C_{O2}}$$

**Figure 5:**
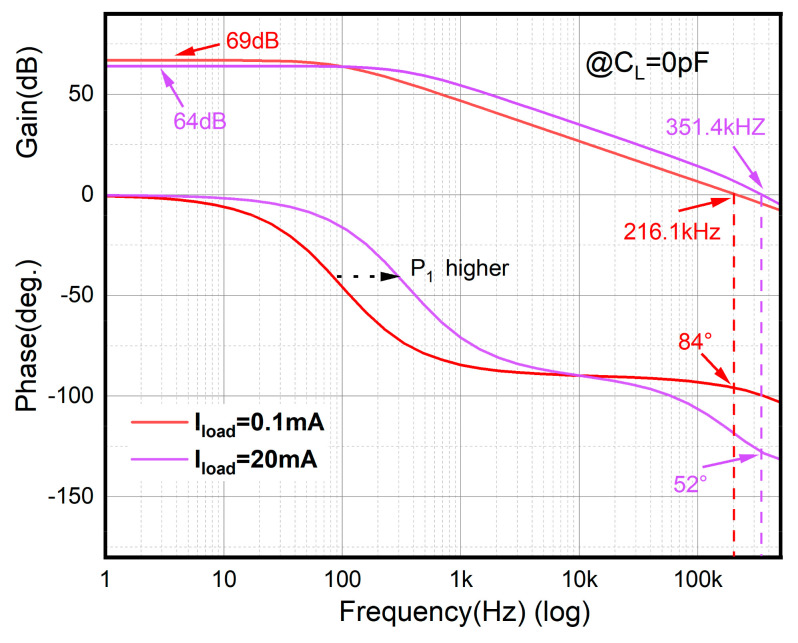
Frequency response at different I_Load_ values.

*P*_1_ is the dominant pole, while *P*_2_ and *P*_3_ are the non-dominant poles. When the load current I_load_ increases, the output resistance decreases due to its inverse proportionality with the load current. Since g_mp_ is proportional to $\sqrt{I_{load}}$ and *R_O_* is proportional to 1/I_load_, *P*_1_ and *P*_2_ are proportional to $\sqrt{I_{load}}$. To guarantee system stability, the phase margin should be above 60°; so, *P*_2_ and *P*_3_ should be placed above the double unity gain frequency under all conditions. The approximate output resistance of EA *R_O_*_2_ = $r_{O24}||r_{O25}$ is several megaohms, while the equivalent capacitor at the input of M_P_ is approximately tens of pF. Without the proposed buffer, the non-dominant pole is generated by the resistance R_O2_ and capacitors C_gs_ and C_gd_, which are near to the dominant pole, thereby leading to stability issues. However, in this paper, the buffer incorporating a low output resistance separates this low-frequency pole into two high-frequency poles. The frequency response when I_load_ changes is shown in Figure 5. The circuit could keep steady when I_load_ rises to 20 mA without an output capacitor.

**Figure 6:**
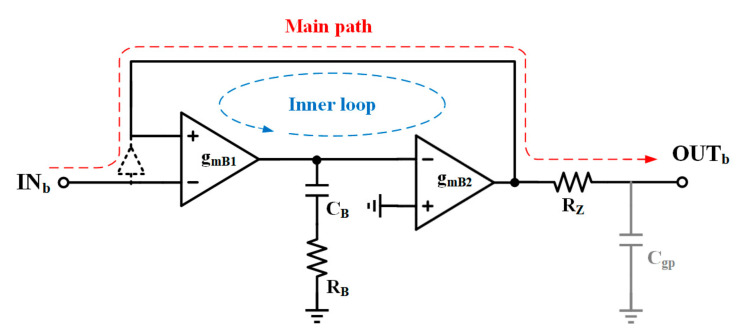
Block diagram of super source follower buffer.

The stability of the entire proposed loop must be ensured under all conditions, along with the buffer stability, which has been approximately replaced by *A_vbuf_*,. To analyze the loop stability of the buffer, the block diagram in Figure 6 is proposed. The resistor *R_Z_* is added to generate a zero with the parasitic capacitor *C_gp_*. The presence of this zero ensures the stable operation of the inner loop, even when *C_gp_* is large and, in turn, generates another pole for the main feedback loop. The capacitor *C_B_* and resistor *R_B_* are also added to compensate. Although the Miller gain applied to *C_B_* is relatively small, it should be noted that one end of *C_B_* is connected to the drain of M_24_. Consequently, to facilitate a simplified analysis within the block diagram, both *C_B_* and *R_B_* are connected in series and grounded. The gain of the inner loop is approximately given by Equation (10).

(10) $$A_{vloop}\approx \frac{g_{m27}r_{o31}\left(1+sR_{B}C_{B}\right)\left(1+sR_{Z}C_{gp}\right)}{\left(1+sr_{o31}C_{B}\right)\left(1+sR_{Z}C_{gp}\right)}$$

### 2.4. Transient Response Analysis

**Figure 7:**
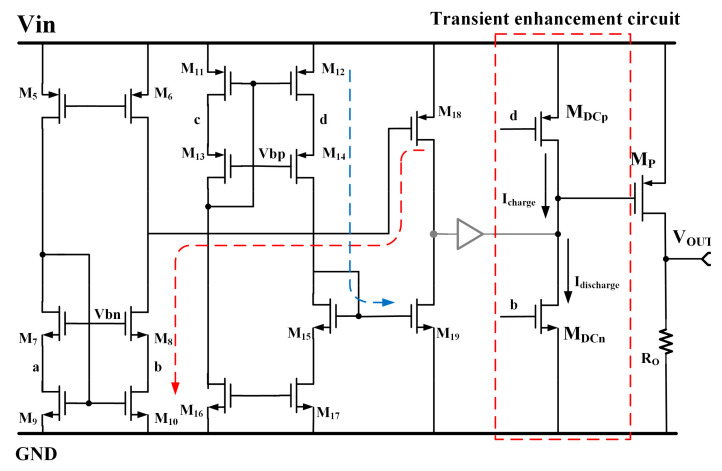
Schematic of transient enhancement circuit.

The proposed CL-LDO is expected to exhibit an enhanced transient response and reduced undershoot and overshoot spikes. To enhance the transient response, the dynamic charging transistors are added to deal with large transient steps. As depicted in Figure 7, the gate of PMOS M_DCp_ and NMOS *M_DCn_* is directly regulated by the voltages V_d_ of the d node and V_b_ of the b node in the folded cascode amplifier. The ratio of the size of *M_DCTp_* to *M_DCTn_* is set as 1:2. At steady state, the *M_DCn_* and *M_DCp_* transistors are biased in the cutoff region by V_b_ and V_d_ because the overdrive voltages of both M_16_ and M_18_ are lower than the threshold voltages of *M_DCn_* and *M_DCp_*. In this case, this transient enhancement circuit will not influence the stability even with some offset at the input pairs. However, when a large transient step occurs, the output voltage will increase or decrease instantly. Since V_b_ and V_d_ are naturally sensitive to the transient response, large current I_charge_ and I_discharge_ can be generated to charge or discharge the large gate parasitic capacitor of M_P_ without additional sensing circuits. The deviation of the output voltage V_dev_ that causes V_b_ and V_d_ to bias dynamic charging transistors in open mode is given in Equations (11) and (12).

(11) $$V_{devp}=\frac{\left|V_{th,MDCTp}\right|-V_{ov12}}{g_{mn}R_{d}}$$

(12) $$V_{devn}=\frac{V_{th,MDCTn}-V_{ov10}}{g_{mp}R_{b}}$$

where *V_th_*_,*MDCTp*_ and *V_th_*_,*MDCTn*_ are the threshold of *M_DCp_* and *M_DCn_*, and where *V_ov_*_12_ and *V_ov_*_10_ are the overdrive voltages of M_12_ and M_10_. Here, *g_mp_* and *g_mn_* stand for the trans-conductance of the input pair consisting of M_1,2_ and M_3,4_, while *R_d_* and *R_b_* denote the equivalent resistance at nodes d and b, respectively. When considering the size of *M_DCp_* and *M_DCn_*, due to the presence of a small parasitic capacitance, the minimum length is used to ensure a rapid response time. According to the equation, it is evident that *V_devp_*_,*n*_ is controlled by the threshold voltage of *M_DCp_*_,*n*_. However, if the transistor’s size and voltages of the b and d nodes are appropriately designed, *V_devp_*_,*n*_ will be constrained by the gain of this transient enhancement circuit and will remain unaffected by *V_th_*_,*MDCp*,*n*_. Additionally, a smaller *V_devp_*_,*n*_ leads to a reduced ΔV_OUT_. To effectively regulate the overshoot and undershoot voltage at one-tenth of V_OUT_, it is recommended that *V_devp_*_,*n*_ be set to approximately 100 mV.

To control the transient response limitation caused by the finite bandwidth of the main linear regulation loop, a simple operational trans-conductance amplifier (OTA) with a constant small current is incorporated to regulate the high impedance node of the proposed SSFB. By employing this simple OTA for control, the unity gain frequency can be pushed to a higher point and the bandwidth of the main loop can be expanded. The loop frequency response of the whole circuit with added OTA is shown in Section 3 to demonstrate the stability.

## 3. Simulation Results and Discussion

The proposed CL-LDO is simulated using a TSMC 0.18 μm standard CMOS process. With a supply voltage range of 1.2 V to 1.8 V and a bias current of 2 µA, this CL-LDO is designed to maintain output voltage regulation at 1 V. We will talk about the precise simulation findings for the stability, load regulation, line regulation, and power supply rejection under various conditions.

### 3.1. Loop Frequency Response

**Figure 8:**
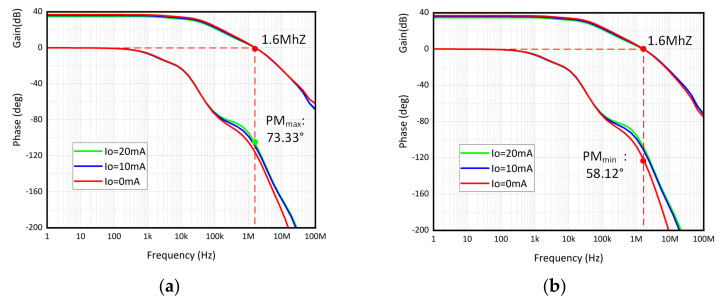
Simulation results of loop frequency response under different I_O_ and (**a**) C_L_ = 0 pF; (**b**) C_L_ = 100 pF.

The loop frequency response under different load capacitor and load current combinations is shown in Figure 8. Figure 8a shows the Bode diagram without load capacitor, while Figure 8b shows the load capacitor at 100 pF. Both (a) and (b) show the current load range from 20 mA to 0 mA. As previously analyzed, the bandwidth is pushed from several hundred kilohertz to 1.6 megahertz. On the contrary, the dc gain decreases by approximately 30 dB, which demonstrates the trade-off between gain and speed. It is evident that the load condition has little influence on stability since the node at output is set as the non-dominant pole. The minimum phase margin is 58.12° when the load current is 0 mA and the load capacitor is 100 pF. Meanwhile, the maximum phase margin is 73.33° when the load current is 20 mA and without a load capacitor. 

### 3.2. Load Transient Response and Load Regulation

**Figure 9:**
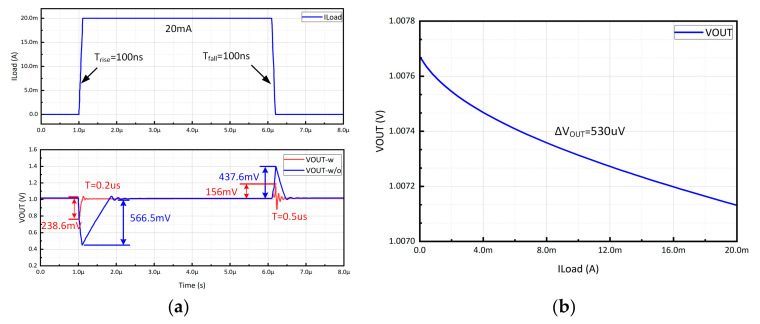
(**a**) Simulated load transient response of the proposed CL-LDO with I_Load_ step between 0 A and 20 mA. (**b**) Load regulation with C_L_ = 0 pF and V_IN_ = 1.8 V.

The load transient response and load regulation of the proposed CL-LDO are depicted in Figure 9. The load current ranges from 0 A to 20 mA, while the rise and fall times of I_Load_ for emulating the load transient response are set at 100 ns. The simulation results of CL-LDO with and without the transient enhancement circuit are compared and illustrated in Figure 9a. The response time of CL-LDO during load current rise and fall is significantly improved, with a reduction to 0.2 µs and 0.5 µs, respectively, surpassing the performance of the circuit without a transient enhancement circuit. The undershoot voltage drops from 566.5 mV to 238.6 mV, and the overshoot voltage drops from 437.6 mV to 156 mV. Figure 9b shows the load regulation when V_IN_ = 1.8 V and C_L_ = 0 pF. The V_OUT_ suffers from a 530 µV variation when I_Load_ changes from 0 to 20 mA, resulting in a load regulation of 26.5 µV/mA. 

### 3.3. Line Transient Response and Line Regulation

**Figure 10:**
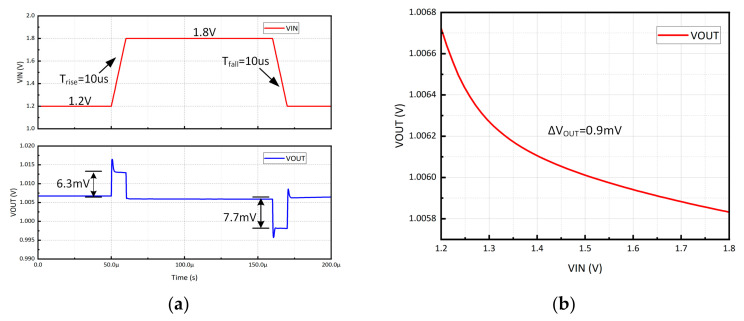
(**a**) Simulated line transient response of the proposed CL-LDO with VDD step between 1.2 and 1.8 V. (**b**) Line regulation with C_L_ = 100 pF and I_O_ = 0 mA.

Figure 10a illustrates the line transient response when the V_IN_ step is between 1.2 V and 1.8 V at an edge time of 10 µs of the proposed CL-LDO. The line transient response is simulated at C_L_ = 100 pF and I_O_ = 0 mA. The output voltage exhibits an overshoot of 6.3 mV when the V_IN_ steps up. Conversely, it experiences an undershoot of 7.7 mV when the line regulation, which quantifies the deviation in output voltage, is simulated under identical conditions. The voltage output, as depicted in Figure 10b, exhibits a variation of 0.9 mV, resulting in a line regulation of 1.5 mV/V.

### 3.4. Power-Supply Rejection

**Figure 11:**
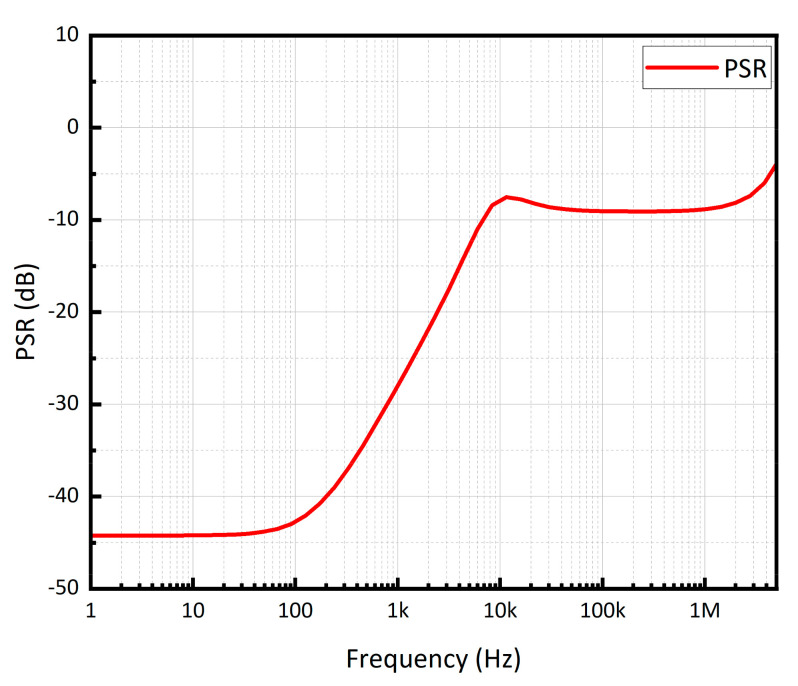
Simulated PSR performance of the proposed CL-LDO.

The *PSR* of an LDO is given in [18], as shown in Equation (13).

(13) $$PSR=\frac{v_{out}\left(s\right)}{v_{in}\left(s\right)}=\frac{\frac{R_{L}}{R_{L}+r_{ds}}}{\left(1+\frac{s}{\omega_{0}}\right)\left(1+LG\left(s\right)\right)}$$

where *LG*(*s*) stands for the loop gain, ω_0_ is the pole at the output of the LDO, and *R_L_* and *r_ds_* denote the load resistance and the output impedance of M_P_, respectively. At low frequency, *PSR* is obviously equal to 1/(1 + *LG*(*s*)). If the *ω*_0_ is the non-dominant pole, the loop gain exhibits a roll-off at −20 dB/decade, resulting in a corresponding decline in the *PSR* at the same rate from the dominant pole. This degradation will persist until the *PSR* remains constant when *LG*(*s*) is significantly smaller than 1. At a higher frequency, the *PSR* is primarily influenced by the load capacitor and the M_P_’s parasitic capacitors, resulting in a reduction in the equivalent resistance. The simulated PSR performance of the proposed CL-LDO at a 20 mA load current and 0 pF load capacitor is shown in Figure 11. The PSR of the proposed CL-LDO is −43 dB at 100 Hz and −9 dB at 1 MHz. The attenuation trend of PSR degrades by −20 dB/decade after the dominant pole, which corresponds to the analysis of Equation (13) and the stability analysis.

### 3.5. Performance Comparison

The figure of merit (*FOM*) in [19,20] is adopted to evaluate the different current efficiencies of the CL-LDOs. The smaller FOM indicates superior performance in terms of the current efficiency and load transient response. The parasitic capacitance of the power transistor is influenced by the minimum channel length (*L*) in different processes. A process with a shorter minimum *L* may result in a smaller *FOM* due to the reduced parasitic capacitance of the transistor. To ensure a fair comparison, the *FOM*_1_ equation, originally proposed in [6] with consideration of the minimum *L*, is used to compare the transient response.

(14) $$FOM_{1}=\frac{T_{edge}\cdot \Delta V_{OUT}\cdot \left(I_{Q}+I_{Load(\min)}\right)}{\Delta I_{Load}\cdot L^{2}}$$

The performance comparison with previously reported CL-LDOs is summarized in Table 1. In this table, the representative study findings from recent years are compared with this design to demonstrate the improved performance. The *I_Q_* row shows that the power consumption of this design is only slightly higher than that proposed in 2020, and significantly lower than other architectures. The Load Reg and T_settle_ rows show that this design can achieve good voltage regulation and a fast response performance when the load current changes. These comparison results demonstrate that even under the relatively backward 180 nm process, the architecture can still have lower power consumption, smaller load regulation and a faster response speed. As a result, this demonstrates a lower FOM_1_, indicating a higher performance benefit.

## 4. Conclusions

This paper proposes a new capacitor-less LDO structure for digital analog hybrid circuits. The proposed capacitor-less LDO utilizes RIPO and SSFB to satisfy the design challenge of stability typically associated with the absence of on-chip capacitors. This proposed structure is stable at a load current range of 0 mA to 20 mA, with a maximum allowable CL of 100 pF. With the transient enhancement circuit, this structure achieves a good transient response while ensuring stability. The settling time is about 0.22 µs when the load current steps from 0 mA to 20 mA within 100 ns.

## Figures and Tables

**Table 1:** Main performance summary and comparison.

Reference	[21]	[22]	[14]	[23]	This Work
**Year**	2017	2018	2020	2022	**2023**
**Process**	40 nm	130 nm	65 nm	350 nm	**180 nm**
**V_IN_ [V]**	1.1	1–1.4	0.95–1.2	2.7–3.3	**1.2–1.8**
**V_OUT_ [V]**	1	0.8	0.8	2.5	**1**
**I_Load,max_ [mA]**	200	40	100	100	**20**
**I_Load,min_ [mA]**	0	9	0	0.1	**0**
**C_L_ [pF]**	0–100	0–50	0–100	0–100	**0–100**
**I_Q_ [µA]**	275	200	14	66	**47**
**Δ** **V_OUT_ [V]**	0.12	0.036	0.23	0.255	**0.15**
**Line Reg [mV/V]**	0.75	0.857	12	0.8	**1.5**
**Load Reg [µV/mA]**	19	248	90	60	**25**
**T_edge_ [ns]**	100	100	220	400	**100**
**T_settle_ [µs]**	0.8	0.04	3.2	1.2	**0.5**
**FOM_1_ [** **ns** ***·*** **V/** **µ** **m^2^]**	10.65	0.62	1.67	0.63	**1.09**

## Data Availability

Data are contained within the article.
